# Protocol of a longitudinal study on the specific needs of Syrian refugee families in Switzerland

**DOI:** 10.1186/s12914-019-0216-4

**Published:** 2019-12-16

**Authors:** Nahema El Ghaziri, Jérémie Blaser, Joëlle Darwiche, Joan-Carles Suris, Javier Sanchis Zozaya, Régis Marion-Veyron, Dario Spini, Patrick Bodenmann

**Affiliations:** 1 Department of Vulnerabilities and Social Medicine, Center for Primary Care and Public Health (Unisanté), University of Lausanne, Rue du Bugnon 44, CH-1011 Lausanne, Switzerland; 2 Family and Development Research Center, Faculty of Social and Political Sciences, Institute of Psychology, University of Lausanne, Lausanne, Switzerland; 3 Department of Epidemiology and Health Systems, Center for Primary Care and Public Health (Unisanté), University of Lausanne, Lausanne, Switzerland; 4 Department of Ambulatory Care, Center for Primary Care and Public Health (Unisanté), University of Lausanne, Lausanne, Switzerland; 5 Swiss National Centre of Competence in Research: LIVES, and Institute of Social Sciences, University of Lausanne, Lausanne, Switzerland

**Keywords:** Family, Mental health, Resettlement program, Syrian refugees

## Abstract

**Background:**

The ongoing Syrian civil war has led to massive population displacements, leading to the reorganization of the asylum policies of several countries. Accordingly, like other European countries, the Swiss government has recently chosen to implement a specific resettlement program. This program is characterized by the fact that the whole nuclear family is granted a work and residence permit upon arrival, and benefits from enhanced integration services. The aim of the present project is to evaluate the effects of the Swiss resettlement program, with a special focus on mental health, while adopting a family perspective.

**Methods:**

The outcomes of 15 Syrian families taking part in this program will be compared to those of 15 Syrian families that came to Switzerland through other means (i.e., following the usual asylum procedure, which is much more stressful and time consuming). Each family member above 8 years old will be invited to participate to a 3-wave longitudinal survey concerning the resettlement process: upon arrival in the collective shelters, six and 12 months later. Questionnaires will be used for the evaluation of participants’ mental health, risk behaviors, general health, romantic relationship, parent-child relationship, family functioning, parentification, social support, and social identities related to group belongingness.

**Discussion:**

The findings of the present project will provide longitudinal information on Syrian refugees. A comprehensive approach will be adopted by screening potential difficulties that the sample may be faced with and potential strengths that participants may rely on. Accordingly, physical and mental health, as well as the quality of family functioning, the feeling of support and of belongingness to different groups will be evaluated.

We will also compare the results of families who had the chance to immigrate through the Swiss resettlement program, to the results of families that did not. This comparison will allow the elaboration of hypotheses regarding adjusted asylum policies. Furthermore, it will enhance our knowledge regarding the impact of displacement on the family system. Indeed, although the role of the family for the well-being of adults and children has been established, surprisingly few studies have adopted this focus in the asylum field.

## Background

Since 2011, Syria has been facing a humanitarian crisis as part of the country’s ongoing civil war. In 2013, the Swiss Confederation decided to help the displaced Syrian population and started the pilot phase of a project called “resettlement” [1]. This pilot phase allowed the accommodation of 500 particularly vulnerable Syrian refugees in eight Swiss cantons, on a period of 2 years (2013–2015). The procedure of the resettlement program is the following: the Office of the United Nations High Commissioner for Refugees (UNHCR) selects refugees (in Lebanon or Jordan), who are then issued a humanitarian permit upon arrival in Switzerland. Refugees involved in the Swiss resettlement program also benefit from several integration measures, among which the presence of coaches speaking Arabic. These coaches have the mission to accompany and assist the refugees during their resettlement journey [2]. Noticeably, the usual asylum application process takes several months before the seeker potentially obtains an equal permit, which is a source of stress and feeling of uncertainty. It also impedes integration, as several aspects of the daily living are obstructed or impossible without an adequate permit (e.g., renting a flat, having a mobile phone subscription or making a legal employment contract).

In March 2015, the Swiss resettlement project was extended to all cantons, with the initial objective of accommodating 3000 additional Syrians over 3 years. In parallel, the European Union started a program of resettlement of migrants arriving at the borders (especially from “hotspots” in Italy and Greece), among which a certain number of Syrians. The Swiss Confederation decided to take part in this program by hosting a part of these migrants. Unlike the resettlement project of the Swiss government, these refugees have to follow the normal path of the asylum application process in Switzerland, but are transported directly from the borders of Europe to Switzerland.

Through these various projects, Switzerland is planning for the arrival of a significant number of Syrian migrants. In order to organize and achieve successful resettlement, acknowledging this population’s specific needs is critical. According to Hassan, Ventevogel, Jefee-Bahloul, Barkil-Oteo, and Kirmayer [3], the on-going conflict may have had several consequences for Syrians’ mental health and well-being. It may both have exacerbated existing issues and created new problems due to violence experiences, multiple losses, and harshness of post migration living conditions. In line with this assumption, multiple studies seem to report a rather high prevalence of post-traumatic stress disorder (PTSD), with 20–83.4% of the participants fitting the diagnosis criteria [4–7]. Syrian refugees also seem to endure various stress and depression symptoms [8, 9]. However, most studies were conducted in bordering countries, such as Turkey or Lebanon, with samples living in camps. Accordingly, little is known regarding Syrian refugees in Europe. Furthermore, to our knowledge no longitudinal study has yet been published. There is therefore a gap in the literature regarding the development of the Syrian refugee’s well-being, once settled in a safe and high-income country.

Regarding the general medical policy of Switzerland, asylum seekers receive care following their arrival in the country. A first screening is performed in order to check for the asylum seekers’ general health, the potential presence or contact with tuberculosis, the existence of addictions, and the experiencing of traumatizing events. The asylum seekers are then sent to their city of residency where the subsequent care procedures are specific to each canton. In Vaud, wherein the present study will be implemented, the Center for Primary Care and Public Health (Unisanté) coordinates the medical care of asylum seekers. The usual procedure is the following: for adults, the hospital provides a medical check-up on arrival, with a vaccine catch-up schedule, serological tests for hepatitis B virus and for HIV. Similarly, children go through a medical check-up and a vaccine catch-up schedule. Additionally, they undergo a Mantoux test. The hospital is also in charge of the medical follow-up of these patients [10]. However, the Swiss resettlement program challenged this procedure. Indeed, as the participants of this program quickly obtain a permit allowing them to live and work in Switzerland, they also quickly exit the asylum seekers process and its associated medical care system. For that reason, a specific healthcare unit had to be developed, which led to the creation of a “family consultation”. Its aim is to provide a medical follow-up similar to that of asylum seekers but with the specificity of following the whole family, in an interdisciplinary framework (collaboration between pediatricians, general practitioners, nurses and community interpreters).

The creation of the family consultation elicited a new perspective on the caring system and highlighted the importance of the family unit for immigrants. It also generated several questions, some of which led to the development of the present project. Indeed, the family consultation represents a unique opportunity to analyze the Syrian refugees’ integration process. In order to obtain a comprehensive picture, information on participants’ physical and mental health, as well as on their family functioning, on their feeling of support and on their feeling of belongingness to social groups will be gathered.

The general goal of this study is to collect longitudinal data on each of the family members of several contingents enrolled in the Swiss resettlement program, and to compare them to a similar number of Syrian families who arrived in Switzerland through other tracks (either through the European resettlement program or by their own means). This should represent approximately 120 persons, and therefore a substantial amount of data, as each family usually includes both parents and more than one child. The study will include adults, but also adolescents and children. Accordingly, the data collected will also represent an opportunity to better understand the effect of an individual’s developmental stage on his or her migration experience. Indeed, each developmental stage is associated with different needs and therefore different difficulties to overcome. For example, children are usually seen as having larger adaptation capacities, which may facilitate their integration [11]. On the other hand, compared to adults, they tend to represent a more vulnerable population and cumulative adversity may have significant consequences for them [12]. Adolescence is a challenging period of life and immigration (because of its causes and/or consequences) may exacerbate the normative crisis that it represents. Surprisingly, several studies indicate that immigrant adolescents tend to display rather good outcomes and even appear to surpass the outcomes of locals, which has been labeled as the “immigrant paradox” [13]. Finally, parenting is also a tough experience. It is associated with additional stress due to the responsibilities that it implies, and immigrant parents seem to go through further emotional difficulties compared to non-parents [14]. All of these features indicate that the immigration journey will not be the same based on one’s developmental stage. Consequently, investigating the role of participants’ age on their outcomes appears to be an interesting avenue to explore.

The results of this study will be useful for advising policy makers concerning the caring services implemented for migrants in Switzerland. In a more general perspective, it will provide information regarding the issues of integration measures. The results will also be useful for prevention interventions and for the promotion of mental health in the migrant population. Furthermore, it will represent a great source of knowledge regarding the effects of forced migration on the family unit. Several authors have highlighted the scarcity of research on the subject [15, 16] and filling this gap would represent a significant add-on to the field, considering the importance of family for parents’ and children’s well-being [17–19].

## Hypotheses and aims

First, we expect a high prevalence of mental disorders. We also assume this hypothesis to be particularly true for the resettlement families who are selected upon vulnerability criteria. Accordingly, the statistics issued from the pilot study of the Swiss resettlement program showed that among 503 refugees, the health of 41% was considered unsatisfactory due to both physical and psychological issues [20].

Second, we presume that the resettlement families will show a less steep deterioration of psychological health across time, compared to the Syrian asylum seekers. Indeed, these families will benefit from a residence and work permit upon arrival, as well as a specific and intensive integration program, which should buffer the usual decline that is often observed among refugees, due to post-migration stressors (e.g., housing conditions and length of asylum procedures) [21, 22]. The confirmation of this hypothesis would represent preliminary information on the benefits of the Swiss resettlement program. On a political level it would provide support to the continuation of this program and help adapting the asylum policy of Switzerland, but also of other western countries hosting Syrians refugees.

Third, we believe that children will display a better development of well-being, compared to adults. This should be true for both groups, as all children will attend school, no matter their legal status. Accordingly, they will all benefit from the strong means of integration that school represents, whereas adults’ access to work and social services will be facilitated for resettlement families. Noticeably, although attending school should attenuate the differences between children of the two groups, we acknowledge that their development should be affected by their parents’ migration process.

Finally, we await psychological health to be positively associated with high quality family functioning [13], feeling of support [23] and feeling of belonging to different groups in Syria, before migration, and in Switzerland, after migration [24]. In particular, we expect the effect of the legal status (being part of the Swiss resettlement project or not) on the psychological health to be moderated by the variables cited above. In other words, we suggest that the positive effect of having a more stable legal situation on mental health will be enhanced if participants are part of high functioning families, feel greatly supported and consider they belong to several social groups. We also presume that these variables will act as protective factors against the detrimental effects of having a less stable legal situation [22]. However, these variables may also have mediating effects, particularly for the psychological health of children. For example, the stressors linked to the family’s legal status might undermine the adults’ parental capacities and the family functioning, therefore hindering the psychological health of the children [25]. For that reason, both moderating and mediating hypotheses will be tested.

The study has four general aims: 1) provide a database regarding the physical and mental health of Syrian refugees and asylum seekers arriving to Switzerland (including both children and adults); 2) compare the development of two groups of Syrian immigrants based on their legal status, with group 1 arriving through the Swiss resettlement project and group 2 arriving through European programs or through their own means; 3) compare the psychological health of the participants based on their age group: childhood (< 10 years old), adolescence (10 to 18 years old), young adulthood (19 to 50 years old), middle adulthood (51 to 65 years old) and retirement (> 65 years old); 4) analyze the development of the participants’ physical and mental health, family functioning, and feeling of support and belongingness to social groups, using three measurement points: upon arrival in the collective shelters of the canton of Vaud, 6 months later and 1 year later.

## Methods

### Design of the Study

The study is longitudinal. Quantitative data will be collected using a standardized questionnaire during face-to-face interviews at three time points, each separated by 6 months. Participants will be seen at their arrival in the canton, in collective shelters (T1). Six months later, a second meeting will be set, corresponding to the period wherein resettlement families should be transferred to individual housings (T2). A last meeting will be set 12 months after T1, in order to evaluate the development of the resettlement families after moving into a home of their own.

A research collaborator speaking Arabic will present the study to the families in the collective shelters and will then see each family individually, at the shelter (T1) or in their own housings (T2, T3). Before starting the research, family members will be asked to sign a consent file, which they can keep. For each participant an oral agreement is required. However, with children below 14 years old, the parents sign the consent file. This age limit was chosen as the capacity to make autonomous decisions is usually considered to be achieved between ages 12 and 16 [26].

### Study populations

Two populations are considered: Syrian refugee families that came to Switzerland through the Swiss resettlement program (1) and families of Syrian asylum seekers that came to Switzerland through European programs or by their own means (2).

Noticeably, the admission criteria fixed by the Swiss Federal Council for the contingents of the resettlement program are the following [2]: an enhanced need for protection; willingness and potential for integration; 40–60% of women and young girls; a rate of aged, ill or disabled individuals of no less than 7%.

To be included in the research, a family unit has to comprise both parents and at least one child. Each group should be composed of 15 families.

### Measures

During the first interview, socio-demographic data will be collected concerning the age, sex, religion, languages spoken, educational level, socio-economic status in Syria, civil status, number and age of children, prior network in Switzerland, pre-migration profession, incarceration in Syria or during the migratory journey, detailed migration itinerary, date of arrival in Switzerland, legal status upon arrival in Switzerland.

The rest of the measures will be collected at each of the three time points and are listed in Table 1.

**Table 1:** Measurement instruments of the research variables and their characteristics

	Questionnaires	Number of items	Target
Risk behaviors	ASSIST [27]	6 items for each of 9 different substances (tobacco, alcohol and drugs)	≥ 14 years
Psychiatric disorders	M.I.N.I., [28]	• Major depressive disorder, 22 items • Panic disorder, 16 items • Post-traumatic stress disorder, 15 items • Generalized anxiety, 11 items	≥ 18 years
	M.I.N.I. kid [29]	• Major depressive disorder, 13 items • Panic disorder, 23 items • Separation anxiety, 18 items • Post-traumatic stress disorder, 15 items	8–17 years
General health	SF-12 [30]	12 items	≥ 14 years
	SF-10 [31]	10 items	0–13 years (filled by mothers)
Romantic relationship quality	S-DAS [32]	7 items	Parents
Family functioning	FAD [33]	12 items	≥ 8 years
Quality of the parent-child relationship	A-FPRQ [34]	13 items	Parents and firstborn child if ≥8 years
Parentification	PI [35]	22 items	Children ≥ 8 years
Support	MSPSS [36]	12 items	≥ 8 years
Group belongingness	GB [37]	4 items (Syria /Switzerland)	≥ 8 years

### Data analysis

In order to test our hypotheses, descriptive analyses will be performed. We will also compare group 1 and group 2 using Wilcoxon-Mann-Whitney tests. Finally, multiple regression analyses will be performed in order to understand what variables have a significant effect on the participants’ psychological health. Regarding the sample size, all of the families entering the canton of Vaud through the resettlement program will be included (during 1 year). It should correspond to approximately 15 families and 120 individuals. In line with this estimation, the comparison group will also be composed of 15 families. As all of the resettlement families are included, there is no need to estimate the power calculation.

### Ethical statements

This study has been approved by the Swiss Ethics Committees (reference number: 2018–00925). Its procedure is not very constraining, as it consists of three meetings of approximately 1 h each. However, discussing potentially painful memories or traumas could be hard on the participants. For that reason, in case of need, specific care will be provided: a psychiatrist will be made available in order to ensure the best management of the participants in need of psychological support.

## Discussion

This project will allow evaluating the impact of the Swiss resettlement program, by comparing the outcomes of families that benefit from it, to the outcomes of families that do not. Accordingly, the results of the study will help formulate hypotheses regarding asylum policies and integration measures. In a broader perspective, the findings will be useful for the development of prevention treatments and the promotion of mental health. However, it should be reminded that the feasibility of this research is directly dependent on the political decisions regarding migration and borders. These decisions may change quickly, implying potential adaptations to the present protocol.

Humanitarian organizations such as the United Nations emphasize the importance of the family system for the care and protection of children in crisis settings [38]. However, in the context of forced migration, few studies have focused on this specific system [15]. It is the novelty of the present project: focusing on families and more specifically, analyzing the development of Syrian families’ psychological health, following exodus to Switzerland. Accordingly, although a lot of work has been done regarding the effects of war, displacement and resettlement on the psychological well-being of adults and children, few is known regarding their effects on family functioning [9, 39–41]. Filling this gap would allow a broader understanding of the impact of forced migration. Indeed, migration is a communal experience: each of the family members must adapt to the situation implying multiple transformations in the family life [42]. Furthermore, while the family system may represent a major resource to overcome hardships it can also represent an additional stressor or a vulnerability factor. For example, when trauma does not allow parents to fully embrace their role toward their children [43]. By collecting longitudinal data from each of the family members, this project will substantially add to our understanding of the refugee/asylum seeker experience. Indeed, the focus of the project is on a whole system instead of individuals, therefore adopting a comprehensive framework and allowing a thorough analysis. Finally, the longitudinal setting will provide information regarding the development of these families at different key points of their migration journey.

## Data Availability

Not applicable.

## Abbreviations

A-FPRQ: Arab family peer relationship questionnaire
ASSIST: Alcohol, smoking and substance involvement screening test
FAD: Family assessment device
M.I.N.I: Mini-international neuropsychiatric interview
MSPSS: Multidimensional scale of perceived social support
PI: Parentification inventory
S-DAS: Short form of the dyadic adjustment scale
SF: Short form
UNHCR: United Nations High Commissioner for Refugees
